# Exploring the shared biomarkers between acute ischemia stroke and diabetes by transcriptome sequencing

**DOI:** 10.12669/pjms.42.5.12759

**Published:** 2026-05

**Authors:** Cong Xu, Yonghong Xu, Guangming Wang

**Affiliations:** 1 Cong Xu School of Clinical Medicine, Dali University, Dali 671000, Yunnan, People’s Republic of China; 2 Yonghong Xu Department of General Surgery, Banan Hospital Affiliated to Chongqing Medical University, Chongqing, Banana 401300, People’s Republic of China; 3 Guangming Wang School of Clinical Medicine, Center of Genetic Testing, Dali University, Dali 671000, Yunnan, People’s Republic of China

**Keywords:** Acute ischemic stroke, Diabetes, Transcriptome sequencing, ceRNA network, Regulatory Mechanisms

## Abstract

**Objectives:**

This study aimed to identify shared genetic signatures and potential molecular mechanisms linking acute ischemic stroke (AIS) and diabetes by analyzing their transcriptomic profiles. We sought to characterize differentially expressed non-coding RNAs (ncRNAs) and mRNAs, elucidate their functional pathways, and construct regulatory networks to uncover cross-talk between these conditions.

**Methodology:**

Peripheral blood samples were collected from five patients with AIS and five patients with diabetes at the First Affiliated Hospital of Dali University between March 1, 2023 to September 31, 2023. Transcriptome sequencing was performed on these blood samples. Differentially expressed miRNAs (n=1,755), mRNAs (n=24,862), lncRNAs (n=7,387), and circRNAs (n=16,413) were identified (fold change >2, FDR <0.05) through comparative analysis of these groups. Functional enrichment was analyzed via GO and KEGG pathways. A ceRNA network integrating miRNA-mRNA-lncRNA-circRNA interactions was reconstructed using Cytoscape, with key regulators validated by degree centrality analysis.

**Results:**

Top dysregulated transcripts included hsa-miR-3614-5p (up) and hsa-miR-935 (down), mRNAs ENSG00000100985/ENSG00000154764 (MMP9/WNT7A), lncRNAs ENSG00000268734/ENSG00000272529, and circRNAs hsa-circ-0027541/hsa-circ-0024837. Pathway analysis revealed: (1) circRNAs modulated T-cell receptor signaling and NK cell cytotoxicity. (2)miRNA-target genes enriched in cancer/stem cell pathways (e.g., PI3K-Akt). (3) mRNAs implicated in inflammatory cascades (TNF/NF-κB). (4)The ceRNA network demonstrated 756 interactions centered on hsa-miR-619-5p, which coordinated mRNA-lncRNA-circRNA cross-talk.

**Conclusion:**

We identified convergent transcriptomic alterations in AIS-diabetes comorbidity, highlighting hsa-miR-619-5p as a hub regulator of immune-metabolic crosstalk. The integrated ceRNA network provides a mechanistic framework linking ncRNAs to inflammatory pathways, offering potential diagnostic biomarkers and therapeutic targets for dual disease management.

## INTRODUCTION

Globally, stroke remains the second leading cause of mortality, while in China it has become the foremost contributor to death and disability since 2015. In 2020, an estimated 17.8 million individuals were affected by stroke in China, with approximately 2.3 million fatalities, a burden exacerbated by an aging population and poorly controlled risk factors such as hypertension, diabetes, and hyperlipidemia.^1^-^4^ Acute ischemic stroke (AIS) impairs cerebral blood flow, leading to neuronal damage through mechanisms including ischemia-induced cell loss, oxidative stress, and neuroinflammation.^5^,^6^

Diabetes mellitus (DM), a chronic metabolic disorder affecting over 463 million people globally in 2019, significantly elevates the risk of ischemic stroke---by approximately 2.27-fold---and worsens post-stroke outcomes.^7^-^13^ The pathogenic link between DM and poor stroke prognosis is primarily driven by hyperglycemia-induced endothelial dysfunction, accelerated atherosclerosis, and enhanced thrombosis. These mechanisms not only increase cerebrovascular vulnerability but also contribute to higher rates of post-stroke mortality, disability, and cognitive impairment.^14^ Given this strong clinical interplay, this study investigates shared molecular and epigenetic mechanisms between AIS and diabetes through transcriptome sequencing. By analyzing co-expression patterns of miRNAs, circRNAs, and lncRNAs across stroke and diabetes groups, we aim to identify non-coding RNA-mediated regulatory networks that may serve as biomarkers for early diagnosis or targets for therapeutic intervention.

## METHODOLOGY

Peripheral blood samples of 5ml each(gathering with the BD PAXgene Blood RNA Tube,QIAGEN of Germany)were collected from five patients with AIS and five patients with diabetes during the same period who were admitted to the First Affiliated Hospital of Dali University from March 1, 2023 to September 30, 2023. Five milliliters of venous blood were collected from each patient. Every patient is satisfied with the AIS or diabetes diagnosis criteria.

### Ethics statement:

The studies involving human participants were reviewed and approved by the Ethics Committee of the Affiliated Hospital of Dali University. The patients/participants provided their written informed consent to participate in this study. Written informed consent was obtained from the individual(s) for the publication of any potentially identifiable images or data included in this article. Ethics review approval number is DFY20220415001; dated April 15, 2022.

### Inclusion & Exclusion Criteria:

This study enrolled patients aged 40-85 with imaging-confirmed acute ischemic stroke within 24 hours of onset and diabetic patients aged 40-80 meeting domestic diagnostic criteria, excluding those with severe organ diseases. Demographic, laboratory, and clinical data were systematically collected.

### RNA Extraction:

To extract blood RNA using the E.Z.N.A.™ PX RNA Kit (omega Bio, USA), 48ml of anhydrous ethanol is first added to RNA wash Buffer II. Remove the sample from the -80^0^C refrigerator and melt at room temperature for two hours. The purity of RNA was detected by Nanodrop 2000® (Thermo Company, USA). The OD260/280 value was 1.7 ~ 2.0, and the OD260/230 value was about 2.5. RNA concentration and integrity were tested with the Agilent 2100 RNA Nano 6000 Assay Kit (Agilent Technologies, CA, USA), where a RIN value of > 7 indicated good integrity. Subsequent experiments required RNA concentration ≥100ng/μl, total amount ≥1μg, OD260/280 value 1.8-2.0, and RIN≥5.8.

### Library construction and transcriptome sequencing:

Transcriptome sequencing was conducted on the Illumina platform, constructing four libraries (miRNA, lncRNA, mRNA, circRNA) from qualified samples. After quality control using FastQC and NGSQC, specific protocols were applied: miRNA libraries were prepared from 18-30 nt RNA fragments; circRNA/lncRNA libraries used Ribo-Zero™ Gold Kits for rRNA depletion. Alignment employed specialized tools: HiSAT2 for lncRNAs, BWA-MEM for circRNAs, and Bowtie for miRNAs. Gene expression was quantified via FPKM, while circRNA expression used the SRPBM method based on junction reads.

### Grouping and Co-expression Analysis:

Gene expression levels were analyzed using R (v4.4.01). Differentially expressed RNAs (circRNA, miRNA, lncRNA, mRNA) were identified with |log_2_FC| ≥ 1 and p < 0.05. Heatmaps and ggplot2 were used for visualization and cluster analysis.

### Functional Enrichment:

GO and KEGG enrichment analyses were performed on target genes with q < 0.05. GO terms covered molecular functions, cellular components, and biological processes. KEGG pathways were analyzed via hypergeometric testing (P < 0.05).

### CeRNA Network Construction:

Differentially expressed lncRNAs and mRNAs were integrated to construct a regulatory network. MiRDeep2 predicted miRNA precursors from lncRNA and circRNA sequences. miRNA-mRNA interaction networks were mapped based on expression differences.

### Statistical Methods:

Categorical data were compared using the Chi-square test. Continuous data were analyzed with SPSS 26.0: t-test for normally distributed data (mean ± SD) and Mann-Whitney U test for non-normal data (median [IQR]). P < 0.05 was considered significant.

### Identification of differentially expressed genes:

To validate RNA-seq results, five randomly selected DEGs (hsa-miR-3614-5p, MMP9, ENSG00000268734, hsa-circ-0027541, hsa-miR-619-5p) were analyzed using RT-qPCR. Total RNA was extracted following the AG21023 kit instructions. cDNA was synthesized using the AG11728 kit according to manufacturer’s protocols. Gene-specific primers (Supplementary Table-SI) were designed via NCBI. PCR reactions were performed, and Ct values were collected using StepOne software. The 2-ΔΔCt method was applied to calculate relative expression changes, using internal reference genes for normalization. Consistency between RT-qPCR and RNA-seq expression profiles for all five genes confirmed the accuracy and reliability of the transcriptome data.

## RESULTS

### Patient clinical data information:

The examination information and data of patients with cerebral infarction and diabetes were collected. The whole blood RNA was extracted from all included peripheral blood samples for the assessment of purity, concentration, and integrity. Subsequently, it was determined that the whole blood RNA from the five patients with AIS and five patients with diabetes met the sequencing criteria for database construction, with the results presented in Table-I.

**Table-I:** The sequencing standard for the construction of the whole blood RNA database.

Cases	Concentration (ng/μL)	Volume (μL)	total quantity (μg)	OD260/280	OD260/230	RINe
A1	266	22	5.9	2.1	1.3	6.7
A2	402	23	9.3	2.1	0.9	7.3
A3	396	22	8.7	2.1	1.0	6.7
A4	350	20	7.0	2.1	0.6	8.6
A5	381	22	8.4	2.1	1.2	8.0
D1	226	22	5	2.1	0.9	7.3
D2	333	22	7.3	2.1	1.3	7.2
D3	252	22	5.5	2.1	1.5	6.9
D4	468	22	10.3	2.2	1.6	7.4
D5	229	22	5	2.1	1.4	7.7

### Differential expression profiles of mRNA, lncRNA, circRNA and miRNA between acute stroke patients and diabetic patients:

**Fig 1:**
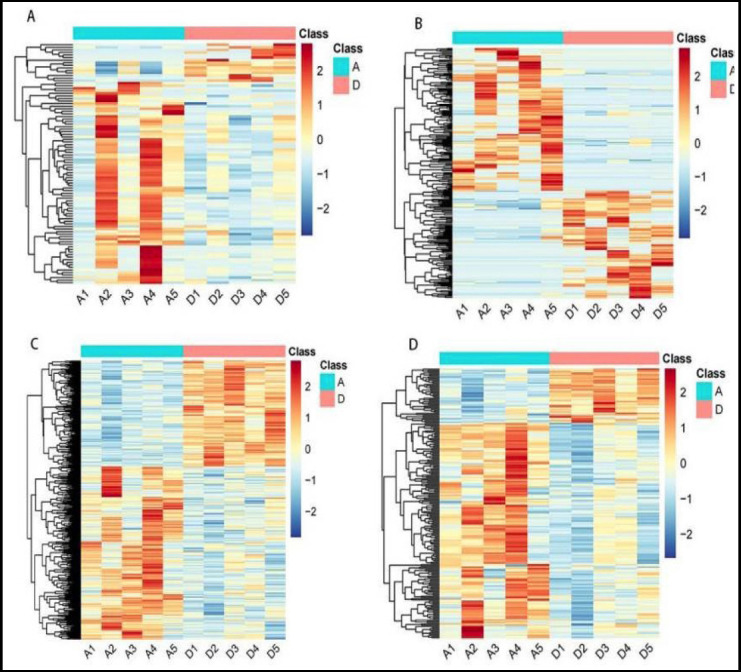
Volcano plot analysis of differentially expressed RNAs (A) miRNA, (B) mRNA, (C) lncRNA, and (D) circRNA expression differences between the acute ischemic stroke (AIS) combined with diabetes group and the control group. Red markers indicate genes with significantly upregulated expression (fold change ≥ 2, p-value < 0.05), green markers indicate genes with significantly downregulated expression (fold change ≤ -2, p-value < 0.05), and gray markers indicate genes with no significant change in expression.

Hierarchical clustering of differentially expressed RNAs was performed using R software, yielding a heatmap (Fig.1). The heatmap displays sample names on the horizontal axis and genes on the vertical axis, with red indicating high expression and blue indicating low expression. Cluster analysis revealed similarities in gene expression among sample groups, suggesting their potential for further study.

Volcano plots illustrate differential expression of miRNA (A), lncRNA (B), circRNA (C), and mRNA (D) between AIS and diabetic groups. Red points represent significantly up-regulated genes (fold change ≥2, p < 0.05), green points indicate down-regulated genes (fold change ≤−2, p < 0.05), and grey points denote non-significant genes.

Differentially expressed RNAs were identified using |log_2_FC| ≥1 and p < 0.05. Based on transcriptome sequencing of peripheral blood samples from five AIS patients and five diabetic patients, the comparisons revealed: 1755 DE miRNAs (837 up, 920 down); most significant: hsa-miR-3614-5p (up) and hsa-miR-935 (down) mRNAs: 24,862 DE mRNAs (12,590 up, 12,549 down); most significant: ENSG00000100985 (up) and WNT7A (down) lncRNAs: 7387 DE lncRNAs (3435 up, 3954 down); most significant: ENSG00000268734 (up) and ENSG00000272529 (down) circRNAs: 16,413 DE circRNAs (8718 up, 7826 down); most significant: hsa-circ-0027541 (up) and hsa-circ-0024837 (down) A co-expression heatmap displays lncRNA, circRNA, miRNA, and mRNA showing differential expression between AIS and diabetic groups.

### Analysis of differentially expressed genes using KEGG and GO pathways:

GO and KEGG pathway analyses were performed to functionally annotate differentially expressed mRNAs, lncRNAs, and circRNAs, using a significance threshold of FDR < 0.05 after p-value adjustment.

For circRNAs, GO analysis revealed enrichment in biological processes (BP) including coenzyme A transmembrane transport, siRNA-mediated gene silencing, positive regulation of renal water transport, beta-glucoside catabolism, regulation of ripoptosome assembly, negative regulation of apoptotic cell clearance, and positive regulation of neuronal action potential. Cellular components (CC) included Toll-like receptor 2/6 complex, presynaptic membrane, rough endoplasmic reticulum, spectrin cytoskeleton, RISC-loading complex, CD40 receptor complex, and SWI/SNF complex. Molecular functions (MF) involved mRNA cap binding, lysine N-acetyltransferase activity, phosphatidylinositol bisphosphate phosphatase activity, siRNA binding, phosphatidylserine binding, alpha-tubulin binding, NF-kappaB binding, and SH3 domain binding.

**Fig.2:**
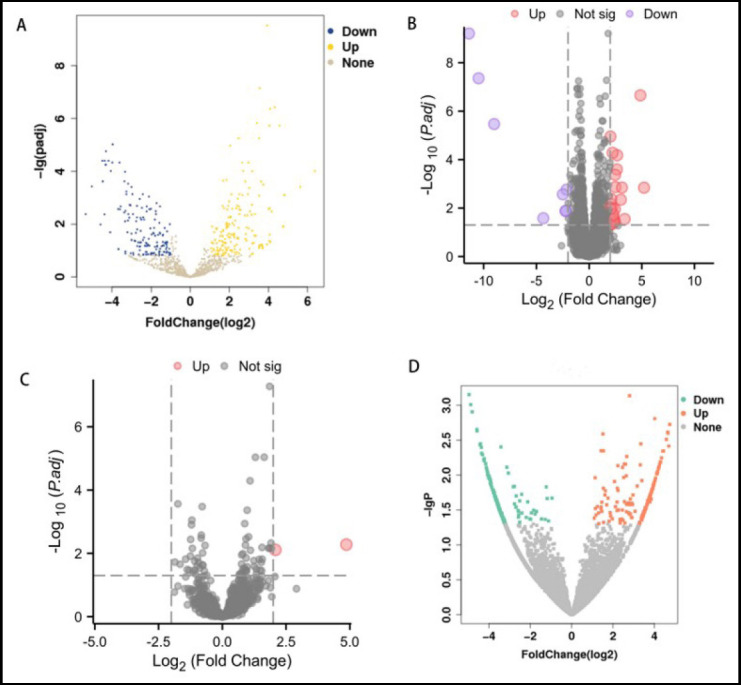
Volcanic plots illustrate the differences in expression levels of miRNA (A), mRNA (B), lncRNA (C), and circRNA (D) between the AIS and diabetes groups. Red markers represent genes with significantly upregulated expression (fold change ≥2 and p-value < 0.05), whereas green markers indicate genes with significantly downregulated expression (fold change ≤−2 and p-value < 0.05). Grey markers signify genes that exhibit no significant changes in expression.

**Fig.3:**
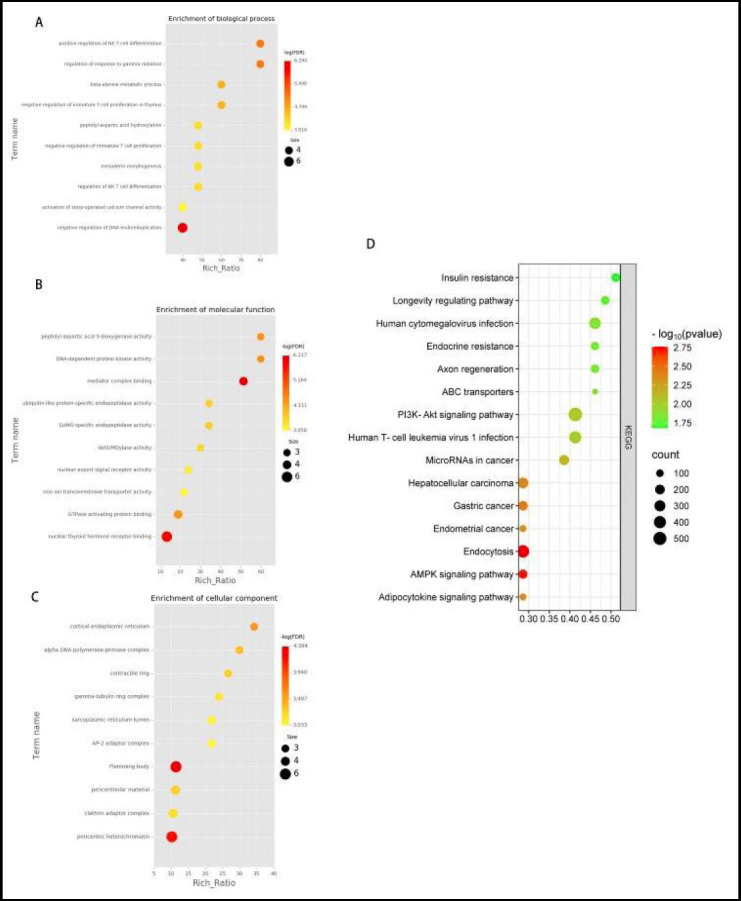
Evaluation of Gene Ontology (GO) and Kyoto Encyclopedia of Genes and Genomes (KEGG) pathways using circRNA expression data. (A) Investigation of gene ontology. Notable GO terms underscore the co-expression patterns at the circRNA level in patients with cerebral infarction and diabetes compared to healthy controls. (B) Analysis of KEGG pathways. This section delves into the fundamental mechanisms driving circRNA co-expression between the cerebral infarction and diabetes group and the control group.

KEGG enrichment analysis (q < 0.05) indicated significant pathways for circRNA-derived genes, including the T cell receptor signaling pathway, natural killer cell mediated cytotoxicity, lysosome, and human T-cell leukemia virus one infection. In the resulting figure, red denotes up-regulated and green down-regulated genes in the KEGG annotation (Fig.2A-3B).

From the GO analysis of known differentially expressed miRNAs, it was found that in BP, it was mainly related to positive regulation of amino acid transport; positive regulation of receptor internationalization; negative regulation of mononuclear cell migration; neuron maturation; regulation of receptor binding; aorta morphogenesis; response to prostaglandin E; positive regulation of antigen receptor-mediated signaling pathway; toll-like receptor nine signaling pathway; coronary vasculature development. In CC, these miRNAs are mainly associated with synaptic cleft; gap junction; postsynaptic density membrane; AMA glutamate receptor complex; ionotropic glutamate receptor complex; neurotransmitter receptor complex; sperm principal piece; sodium channel complex; postsynaptic specialization membrane; dendrite cytoplasm. In MF, these genes are mainly associated with transmembrane-ephrin receptor activity; calcium-activated potassium channel activity; oxidoreductase activity, acting on the CH-NH2 group of donors, oxygen as acceptor; ionotropic glutamate receptor activity; oxidoreductase activity, acting on NAD(P)H, oxygen as acceptor; phosphatidylinositol phosphate kinase activity; G-protein alpha-subunit binding; extracellular matrix structural constituent conferring tensile strength; calcium activated cation channel activity.

Through enrichment analysis of miRNA-derived genes, the KEGG pathway with q value less than 0.05 was calculated and screened, and its distribution map was shown, which was mainly related to Protein processing in endoplasmic reticulum; Proteoglycans in cancer ;Proximal tubule bicarbonate reclamation; Rap1 signaling pathway; Regulation of actin cytoskeleton ;Regulation of lipolysis in adipocytes; Relaxin signaling pathway ;Renin−angiotensin system ;Renin secretion ;RIG−I−like receptor signaling pathway; Salivary secretion; Selenocompound metabolism ;Serotonergic synapse ;Signaling pathways regulating pluripotency of stem cells ;Small cell lung cancer ;Sphingolipid metabolism ;Th17 cell differentiation ;Th1 and Th2 cell differentiation (Fig.3A-3B).

**Fig.4:**
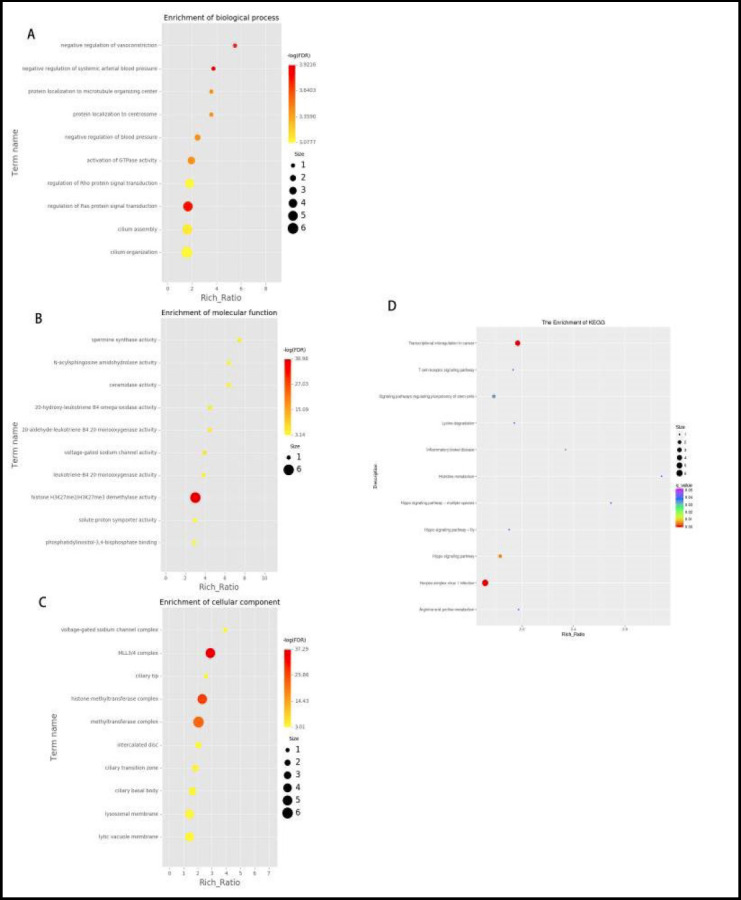
GO and KEGG enrichment analysis based on circRNA-associated genes. (A)-(C) Gene Ontology (GO) analysis. Significant GO terms reveal the biological functions of circRNA-associated genes in patients with cerebral infarction and diabetes compared to healthy controls. (B) KEGG pathway analysis. This part identifies the key signaling pathways potentially regulated by circRNAs in the cerebral infarction with diabetes group versus the control group.

GO analysis of differentially expressed mRNAs revealed significant enrichment in biological processes including regulation of neutrophil-mediated bacterial killing, dIT catabolic and metabolic processes, neutrophil aggregation, detection of and response to triacyl bacterial lipopeptide, TLR1:TLR2 signaling pathway, as well as epithelial and hepatocyte proliferation in liver morphogenesis. Cellular components were primarily associated with granulocyte macrophage colony-stimulating factor receptor complex, Toll-like receptor complexes, phagolysosome, AIM2 and IPAF inflammasome complexes, transcription factor AP-1 complex, haptoglobin-hemoglobin complex, pinosome, and peptidase inhibitor complex. Molecular functions included type I interferon receptor activity, XTP binding, dID phosphatase activity, C5a receptor activity, phosphatidylserine floppase activity, various amine oxidoreductase activities, and organic cation transporter activity. KEGG pathway analysis (q < 0.05) identified significant enrichment in inflammatory bowel disease, leishmaniasis, malaria, neutrophil extracellular trap formation, NF-κB signaling, NOD-like receptor signaling, osteoclast differentiation, and TNF signaling pathway (Fig.4A-4B).

**Fig.5:**
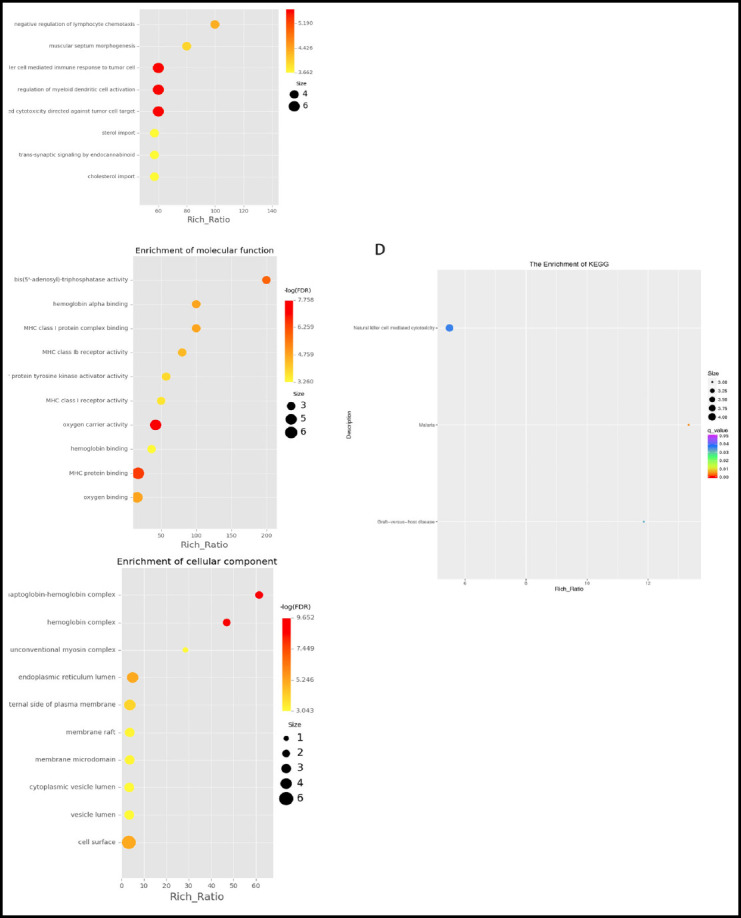
GO and KEGG enrichment analysis based on mRNA expression data.(A)-(C) Gene Ontology (GO) analysis. Enriched GO terms highlight the functional categories of differentially expressed mRNAs in cerebral infarction patients with diabetes compared to healthy controls.(D) KEGG pathway analysis. This section elucidates the major pathways involved in the pathogenesis of cerebral infarction with diabetes based on mRNA expression profiles.

GO analysis of differentially expressed lncRNAs revealed enrichment in biological processes including diadenosine pentaphosphate and polyphosphate catabolism, diadenosine hexaphosphate metabolism and catabolism, adenosine 5’-(hexahydrogen pentaphosphate) metabolism and catabolism, diphosphoinositol polyphosphate metabolism, detection of diacyl bacterial lipopeptide, and positive regulation of response to macrophage colony-stimulating factor. Cellular components were primarily associated with the THO complex and its role in the transcription export complex, specific granule and its lumen and membrane, nucleosome, DNA packaging complex, platelet alpha granule lumen, tertiary granule membrane, and protein-DNA complex. Molecular functions were mainly related to protein heterodimerization activity (Fig.5A-5B).

### Examination of the differential gene co-expression network:

**Fig.6:**
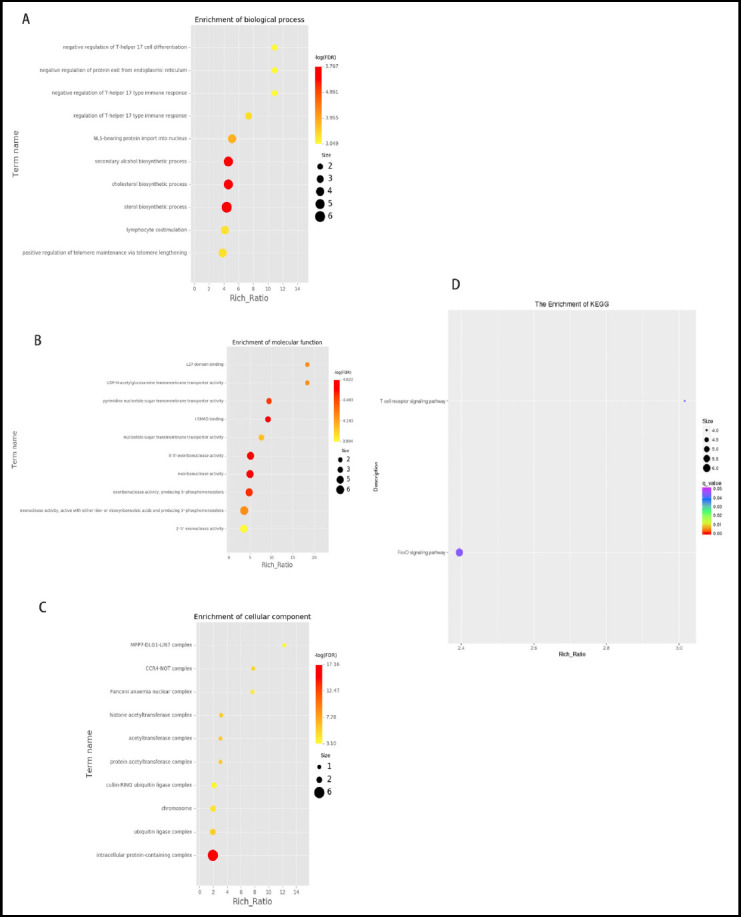
GO and KEGG pathway analysis based on lncrna results. (A) Genetic ontology examination. Major gene ontology term indicating lncRNA co-expression in cerebral infarction patients with diabetes mellitus and healthy subjects. (B) KEGG pathway analysis. To explain the main mechanism of lncRNA co-expression in patients with Cerebral infarction combined with diabetes group and normal group.

The top 150 lncRNA-miRNA interactions were visualized using Cytoscape and ranked by node degree. lncRNAs can sequester miRNAs to regulate their activity, and conversely, miRNAs can bind lncRNAs and influence their function. Studying their interactions provides deeper insight into both molecules. In this network, 11 upregulated miRNAs correlated with 156 downregulated lncRNAs, while four downregulated miRNAs correlated with 261 upregulated lncRNAs. The largest hub, hsa-miR-619-5p, was connected to 358 lncRNAs, suggesting extensive lncRNA-mediated modulation of this miRNA to regulate cellular functions (Fig.6A).

Circular RNAs (circRNAs) can bind to miRNAs and mutually regulate their functions. Investigating miRNA binding sites in differentially expressed circRNAs helps elucidate their roles. Based on miRNA binding site predictions, 40 circRNA-miRNA pairs were identified, among which hsa-miR-619-5p showed the highest connectivity with 33 circRNAs, suggesting these circRNAs play important roles in diabetes and acute ischemic stroke (AIS) (Fig.6B).

The top 150 lncRNA-mRNA interactions were visualized using Cytoscape and ranked by node degree. lncRNAs and mRNAs can bind and reciprocally regulate each other’s functions, with lncRNAs also capable of sequestering mRNAs. Studying these interactions enhances understanding of their regulatory mechanisms. The largest network hub, MSTRG.81445, was connected to 12 lncRNAs (Fig.6C).

**Fig.7:**
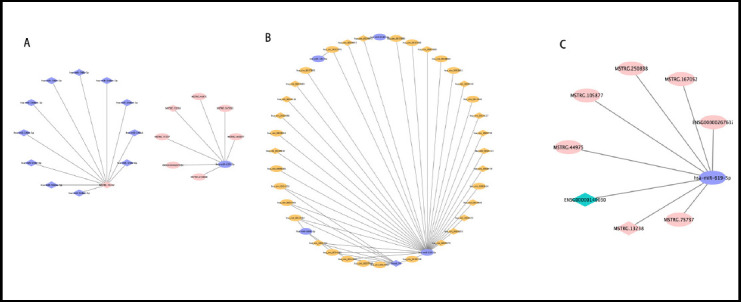
(A): lncRNA and miRNA regulatory network related to Cerebral infarction combined with diabetes group and normal group. (Triangle: down-regulation, Square: up-regulation, purple: miRNA, pink: lncRNA) (B) miRNA and circRNA regulatory network related to Cerebral infarction combined with diabetes group and normal group. (Triangle: down-regulation, Square: up-regulation, purple: miRNA, yellow: circRNA) (C) LncRNA-miRNA-mRNA regulatory network related to Cerebral infarction combined with diabetes group and normal group. (Triangle: down-regulation, Square: up-regulation, purple: miRNA, green: mRNA, pink: lncRNA).

The top 150 miRNA-mRNA interactions were analyzed using Cytoscape. miRNAs and mRNAs can mutually regulate each other’s function through binding, and lncRNAs may further modulate mRNA activity by interacting with them. Studying these integrated interactions enhances understanding of their regulatory pathways and functional roles in biological processes. The largest network hub, hsa-miR-6847-5p, was connected to 398 mRNAs. (Fig.7A).

**Fig.8:**
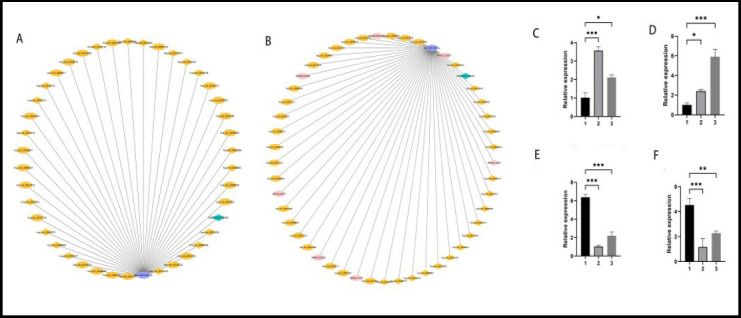
(A): CircRNA-miRNA-mRNA regulatory network related toCerebral infarction combined with diabetes group and normal group. (Triangle: down-regulation, Square: up-regulation, purple: miRNA, yellow: circRNA,green: mRNA) (B) MRNA-miRNA-lncRNA-circRNA regulatory network related to Cerebral infarction combined with diabetes group and normal group. (Triangle: down-regulation, Square: up-regulation, purple: miRNA, yellow: circRNA,green: mRNA;pink: lncRNA) (C) RT-qPCR results of hsa-miR-26b-5p in clinical samples. (D) RT-qPCR results of ENSG00000274012 in clinical samples. (E) RT-qPCR results for hsa-circ-0017780 in clinical samples. (F) RT-qPCR results for ENSG00000267632 in clinical samples.

A total of 2056 up-regulated and 760 down-regulated RNAs were identified. The top 150 interaction pairs by degree value were visualized using Cytoscape. Notably, hsa-miR-619-5p emerged as the largest network hub, connected to 756 target pairs. (Fig.7B).

### DEGs verification was performed by RT-qPCR:

To validate the RNA-seq results, RT-qPCR was performed on five significantly upregulated DEGs: hsa-miR-3614-5p, MMP9, ENSG00000268734, hsa-circ-0027541, and hsa-miR-619-5p. Each gene showed significantly different expression between the diabetic and ischemic stroke groups (Fig.7 C-F, P < 0.05). The RT-qPCR results were consistent with the RNA-seq data, confirming the reliability and accuracy of the identified co-expressed genes.

## DISCUSSION

Acute ischemic stroke (AIS) and diabetes are interconnected pathologies, with diabetes significantly elevating cerebrovascular risk and exacerbating brain injury post-stroke.^15^-^19^ In this study, we identified a total of 1,755 miRNAs, 24,862 mRNAs, 7,387 lncRNAs, and 16,413 circRNAs that were differentially expressed between AIS patients with diabetes and healthy controls. Among these, hsa-miR-3614-5p, MMP9, ENSG00000268734, hsa-circ-0027541, and hsa-miR-619-5p were validated by RT-qPCR, confirming the reliability of our sequencing data.Our findings are consistent with previous studies reporting dysregulated non-coding RNAs in stroke and diabetes. For instance, miR-30c-2-3p has been implicated in metabolic dysfunction, while miR-143-5p and miR-128-1-5p are involved in tissue repair and inflammatory responses.^20^-^23^ Similarly, TNFRSF12A-mediated pathways have been associated with both cerebral ischemia/reperfusion injury and diabetic neuropathy severity^24^,^25^, supporting our observation of shared molecular mechanisms between these two conditions.Non-coding RNAs (lncRNAs, circRNAs, miRNAs) are pivotal regulators in cardiovascular diseases like coronary artery disease and atherosclerosis. They influence processes such as coronary vessel development, calcium/potassium channel activity, and key signaling pathways including MAPK and PI3K-Akt.^26^-^33^ For instance, circACSL1 exacerbates myocardial injury by sponging miR-8055 to upregulate MAPK, while lncRNA ENST00113 and MIAT promote vascular cell proliferation via the PI3K/Akt pathway.^34^,^35^ Our KEGG enrichment analysis revealed that circRNA-associated genes were significantly enriched in the T cell receptor signaling pathway and natural killer cell mediated cytotoxicity, while miRNA-target genes were enriched in cancer/stem cell pathways (e.g., PI3K-Akt) and mRNA-associated genes were enriched in inflammatory cascades (TNF/NF-κB). These findings align with previous reports linking these pathways to both stroke and diabetes pathophysiology.^36^-^39^

Compared to international studies, our results show both similarities and differences. For example, a study by Zhang et al. (2021) reported that circACSL1 aggravates myocardial injury via the MAPK pathway in a European cohort^29^, which is consistent with our findings. However, while some studies have emphasized the role of the NF-κB pathway in stroke patients without diabetes^33^,^34^, our data suggest that this pathway is particularly enriched in patients with comorbid diabetes, indicating potential disease-specific regulatory mechanisms. These discrepancies may be attributed to differences in ethnic backgrounds, sample sizes, disease stages, or methodological approaches.Regionally, our findings are comparable to those from other Asian populations. A study from China by Wang et al. (2020) identified similar lncRNA expression patterns in coronary artery disease patients^32^, supporting the generalizability of our results within Asian cohorts. However, variations in the specific hub genes identified (e.g., hsa-miR-619-5p in our study vs. other miRNAs in different populations) may reflect population-specific genetic backgrounds or environmental factors.This study adds several important contributions to the existing medical literature. First, to our knowledge, this is the first comprehensive transcriptome sequencing study to simultaneously profile four types of RNAs (miRNA, mRNA, lncRNA, and circRNA) in patients with AIS and diabetes comorbidity. While previous studies have focused on individual RNA types or single diseases^20^-^25^, our integrated approach provides a more complete picture of the complex regulatory networks underlying this comorbidity.Second, we identified hsa-miR-619-5p as a novel central hub in the ceRNA network, connecting to 358 lncRNAs, 33 circRNAs, and 756 mRNA targets. Although miR-619-5p has been previously reported in cancer studies42, its role as a master regulator in stroke-diabetes comorbidity has not been described. This finding expands our understanding of non-coding RNA interactions in metabolic and cerebrovascular diseases and provides a new direction for mechanistic studies.Third, our pathway analysis revealed that circRNA-associated genes were enriched in immune-related pathways (T cell receptor signaling, NK cell cytotoxicity), while mRNA-associated genes were enriched in inflammatory pathways (TNF/NF-κB). This suggests that different RNA types may regulate distinct but complementary biological processes in AIS-diabetes comorbidity—a nuanced view that has not been previously reported.The clinical relevance of our findings is twofold. From a diagnostic perspective, the panel of five validated genes (hsa-miR-3614-5p, MMP9, ENSG00000268734, hsa-circ-0027541, hsa-miR-619-5p) may serve as potential biomarkers for early detection of AIS in diabetic patients or for identifying diabetic patients at higher risk of stroke. From a therapeutic standpoint, targeting hsa-miR-619-5p or its downstream networks could offer a novel strategy for simultaneously managing both conditions, potentially improving outcomes in this high-risk patient population.This study integrates the roles of diverse ncRNAs across multiple pathways to construct a systematic regulatory network linking ncRNAs to cardiovascular phenotypes. By analyzing interactions among mRNAs, lncRNAs, circRNAs, and miRNAs, we highlight their synergistic actions, offering a novel perspective for understanding complex cardiovascular and metabolic diseases. Through integrated analysis, we constructed a ceRNA network and identified hsa-miR-619-5p as a central hub with extensive connections to 358 lncRNAs, 33 circRNAs, and 756 mRNA targets. This suggests that hsa-miR-619-5p may serve as a key regulator of immune-metabolic crosstalk in AIS patients with diabetes. Key molecules such as ANRIL, circACSL1, and miR-30c-5p emerge as potential biomarkers or therapeutic targets. For example, targeting lncRNA ENST00113 or circACSL1 could simultaneously intervene in both PI3K/Akt and MAPK signaling, providing dual benefits in attenuating atherosclerosis or myocardial injury.^38^-^40^***Strengths of the study:*** The strengths of this work include comprehensive coverage of multiple ncRNA types and pathways, supported by omics data and preliminary experimental validation. Moreover, the integrated ceRNA network approach provides a systems-level view of the molecular interactions underlying AIS-diabetes comorbidity, which may serve as a foundation for future mechanistic studies and therapeutic development.

### Limitations:

However, limitations remain, including a lack of in-depth functional verification in specific cell or animal models, variability in sample sources and disease stages across studies, and limited understanding of ncRNA roles in neurotransmitter receptor complexes or ion channel activity. Additionally, the relatively small sample size (n=5 per group) may limit the statistical power and generalizability of our findings. Future studies with larger cohorts and functional experiments are needed to validate these results.This study elucidates the critical roles of the PI3K/Akt and MAPK signaling pathways in linking diabetes and ischemic stroke, where they regulate inflammation, metabolism, oxidative stress, apoptosis, and vascular homeostasis.^40^-^42^ MAPK activation exacerbates neuroinflammation and blood-brain barrier disruption post-stroke^43^,^44^, while dysregulation of these pathways contributes to metabolic dysfunction. Through integrated analysis, a complex ceRNA network was constructed, identifying hsa-miR-619-5p as a central hub with extensive connections to lncRNAs, circRNAs, and mRNAs. These findings provide systematic molecular insights into cardiovascular-metabolic comorbidity, offering a theoretical basis for biomarker discovery and targeted therapy development, despite limitations in sample size and need for functional validation.

## CONCLUSION

The findings suggest that mRNAs, lncRNAs, circRNAs, and miRNAs may play functional roles in the development and interplay of AIS and diabetes. Advances in gene sequencing now allow deeper exploration of their epigenetic relationships and biological pathways, providing substantial theoretical and data support for early diagnosis, personalized treatment, and improved patient prognosis. By analyzing interactions among these RNAs, this study aims to clarify the complex connections between diabetes and AIS, offering a scientific foundation for more effective clinical strategies.

### Author Contributions:

**CX and YX:** Data curation, visualization, writing—original draft, software.

**GW:** Resources. CX and GW: Writing—review & editing.

**GW and YX:** are the guarantors of this work and, as such, had full access to all the data in the study and take responsibility for the integrity of the data and the accuracy of the data analysis.All authors contributed to the writing of the manuscript, read and approved the final version of the manuscript.

## Data availability statement:

The datasets generated and/or analyzed during the current study are available from the corresponding author on reasonable request.
